# Synthesis and Biological Evaluation of O-[3-^18^F-fluoropropyl]-****α****-methyl Tyrosine in Mesothelioma-Bearing Rodents

**DOI:** 10.1155/2013/460619

**Published:** 2013-07-09

**Authors:** I-Hong Shih, Fan-Lin Kong, Mohammad S. Ali, Yinhan Zhang, Dong-Fang Yu, Xudong Duan, David J. Yang

**Affiliations:** Department of Experimental Diagnosis Imaging, Unit 59, The University of Texas MD Anderson Cancer Center, 1515 Holcombe Boulevard, Houston, TX 77030, USA

## Abstract

Radiolabeled tyrosine analogs enter cancer cells via upregulated amino acid transporter system and have been shown to be superior to ^18^F-fluoro-2-deoxy-D-glucose (^18^F-FDG) in differential diagnosis in cancers. In this study, we synthesized O-[3-^19^F-fluoropropyl]-**α**-methyl tyrosine (^19^F-FPAMT) and used manual and automated methods to synthesize O-[3-^18^F-fluoropropyl]-**α**-methyl tyrosine (^18^F-FPAMT) in three steps: nucleophilic substitution, deprotection of butoxycarbonyl, and deesterification. Manual and automated synthesis methods produced ^18^F-FPAMT with a radiochemical purity >96%. The decay-corrected yield of ^18^F-FPAMT by manual synthesis was 34% at end-of-synthesis (88 min). The decay-corrected yield of ^18^F-FPAMT by automated synthesis was 15% at end-of-synthesis (110 min). ^18^F-FDG and ^18^F-FPAMT were used for *in vitro* and *in vivo* studies to evaluate the feasibility of ^18^F-FPAMT for imaging rat mesothelioma (IL-45). *In vitro* studies comparing ^18^F-FPAMT with ^18^F-FDG revealed that ^18^F-FDG had higher uptake than that of ^18^F-FPAMT, and the uptake ratio of ^18^F-FPAMT reached the plateau after being incubated for 60 min. Biodistribution studies revealed that the accumulation of ^18^F-FPAMT in the heart, lungs, thyroid, spleen, and brain was significantly lower than that of ^18^F-FDG. There was poor bone uptake in ^18^F-FPAMT for up to 3 hrs suggesting its *in vivo* stability. The imaging studies showed good visualization of tumors with ^18^F-FPAMT. Together, these results suggest that ^18^F-FPAMT can be successfully synthesized and has great potential in mesothelioma imaging.

## 1. Introduction

Numerous studies have demonstrated that growing cancer cells have higher metabolism of glucose and amino acids than other cells in the body. One well-known modality for imaging the metabolic activity of cancers is positron emission tomography (PET) using ^18^F-2-fluoro-2-deoxy-D-glucose (^18^F-FDG), the current gold standard for cancer diagnosis [1]. However, ^18^F-FDG has limitations such as poor differentiation between low-grade tumor and normal tissues in brain [2] and between tumor and inflamed or infected tissues [3]. Radiolabeled amino acids offer higher specificity in characterizing tumors than ^18^F-FDG does. In particular, radiolabeled aromatic amino acids are attractive alternatives to ^18^F-FDG because of easier chemistry alteration and their ability of detection of upregulated amino acid transporters [4], which indirectly reveal cell proliferation. Therefore, ^11^C- and ^18^F-labeled amino acid analogs were developed as alternative metabolic imaging tracers for PET.

^11^C-methyl methionine (^11^C-MET) and L-1-^11^C tyrosine (^11^C-TYR) have been commonly used for clinical research and practices. Unfortunately, the half-life of ^11^C is only 20 min, and, therefore, ^11^C-labeled amino acid analogs require an inconvenient on-site synthesis which reduces their broad clinical usages. ^18^F has a half-life of 110 min, and it can be used at a centralized remote facility to synthesize radiolabeled compounds which can then be delivered to different hospitals simultaneously. Moreover, low *β* ^+^-energy of ^18^F causes a short positron linear range in tissue, thereby providing high resolution in PET images. A number of ^18^F-labeled amino acid analogs in PET have been investigated, including L-2-^18^F-fluorotyrosine (^18^F-TYR) [5], O-2-^18^F-fluoroethyl-L-tyrosine (^18^F-FET) [6], and L-3-^18^F-fluoro-*α*-methyl tyrosine (^18^F-FAMT). Recently, Wiriyasermkul et al. found that, unlike ^18^F-TYR, ^18^F-FET, and other ^18^F-labeled amino acids, ^18^F-FAMT is transported into cells through L-type amino transporter 1, which contributes to its highly tumor-specific accumulation [4]. ^18^F-FAMT was first studied as a brain-imaging probe [7]; later, its use in detecting oral squamous cell carcinoma [8], nonsmall cell lung cancer [9], and esophageal squamous cell carcinoma [10] was investigated. However, the yield of ^18^F-labeled amino acids by an electrophilic fluorination reaction is low (17% for ^18^F-TYR [5]; 20% ± 5.1% for ^18^F-FAMT [11]). Wester et al. synthesized O-2-^18^F-fluoroethyl-L-tyrosine (^18^F-FET) by a nucleophilic fluorination reaction in about 50 min with an overall radiochemical yield of 40% and evaluated it as a PET tracer for cerebral and peripheral tumors [6]. Hamacher and Coenen synthesized ^18^F-FET using one-pot reaction, and the radiochemical yield obtained within 80 min was about 60% [12]. However, both methods require high-performance liquid chromatography (HPLC) for purification, which limits the possibility of automated synthesis. Wang et al. obtained ^18^F-FET by direct nucleophilic fluorination reaction of the protected precursor N-butoxycarbonyl-(O-(2-tosyloxyethyl))-L-tyrosine methyl ester, followed by a rapid removal of the protecting group, and a labeled intermediate was separated out with Sep-Pak silica plus cartridge [13]. The radiochemical yield was about 40% at the end of synthesis (50 min). Bourdier et al. used this method for automated radiosynthesis of ^18^F-FET, and the yield was about 35% within 63 min [14]. ^18^F-FET was widely used in clinical studies in patients with high-grade or low-grade glioma [15, 16].

Despite the very promising clinical results of ^18^F-FAMT, existing methods for synthesizing ^18^F-FAMT produce a low chemical yield, which limits the availability of the compound for clinical use, and they require high-performance liquid chromatography (HPLC) for purification, which precludes the use of an automated module to synthesize ^18^F-FAMT. Therefore, it is desirable to develop an ^18^F-FAMT analog with high chemical yield that can be applied clinically in most major medical facilities. In the present study, we synthesized unlabeled O-[3-^19^F-fluoropropyl]-*α*-methyl tyrosine (^19^F-FPAMT) and ^18^F-labeled O-[3-^18^F-fluoropropyl]-*α*-methyl tyrosine (^18^F-FPAMT) by using nucleophilic substitution to place a fluorine atom on the aliphatic chain of *α*-methyl tyrosine and solid-phase extraction (SPE) column to purify the products. We then used our customized, fully automated synthesis module to synthesize ^18^F-FPAMT. Finally, we used a rat mesothelioma model to investigate the feasibility of using ^18^F-FPAMT as a tumor-seeking imaging agent.

## 2. Materials and Methods

### 2.1. General

All chemicals and solvents were obtained from Sigma-Aldrich (St. Louis, MO, USA). Nuclear magnetic resonance (NMR) spectra were obtained using a Bruker 300 MHz Spectrometer (Bruker BioSpin Corporation, Billerica, MA, USA), and mass spectra were recorded on a Waters Q-TOF Ultima mass spectrometer (Waters, Milford, MA, USA) at the Chemistry Core Facility at The University of Texas MD Anderson Cancer Center (Houston, TX, USA). An HPLC system (Waters) was integrated with an ultraviolet detector and a flow-count radio-HPLC detector (BioScan Inc., Washington, DC, USA). The analyses of radio-thin layer chromatography (TLC) were performed on radio-TLC Imaging Scanner (BioScan, Inc.). The scintigraphic imaging studies were processed on microPET (Siemens Medical Systems, Inc., Malvern, PA, USA).

### 2.2. Synthesis of N-t-butoxycarbonyl-O-[3-tosylpropyl]-*α*-methyl Tyrosine Ethyl Ester

N-t-butoxycarbonyl-O-[3-hydroxypropyl]-*α*-methyl tyrosine ethyl ester, which we used as the precursor compound for synthesis of ^19^F-FPAMT and ^18^F-FPAMT, was prepared as described previously [17]. Briefly, N-t-butoxycarbonyl-O-[3-hydroxypropyl]-*α*-methyl tyrosine ethyl ester (490 mg; 1.28 mmol) in anhydrous pyridine (32 mL) was cooled to 0°C. Paratoluenesulfonyl chloride (1015 mg; 5.32 mmol) was added to this solution, and the solution was stirred for 30 min. The reaction mixture was then stored in a refrigerator overnight. The mixture was filtered, and the filtrate was poured into an ice and water mixture and extracted with diethyl ether. The ethereal solvent was washed with 30 mL of hydrochloric acid and water (1 : 1, v/v) to remove pyridine, and the solvent was dried over anhydrous MgSO_4_. After filtration and solvent evaporation, N-t-butoxycarbonyl-O-[3-tosylpropyl]-*α*-methyl tyrosine ethyl ester was purified by column chromatography using a silica gel column and eluted with hexane and ethyl acetate (2 : 1, v/v) to yield 430 mg (62.5%). NMR and mass spectrometry were performed to confirm the structures.

### 2.3. Synthesis of**** ^19^F-FPAMT

**Figure 1:**
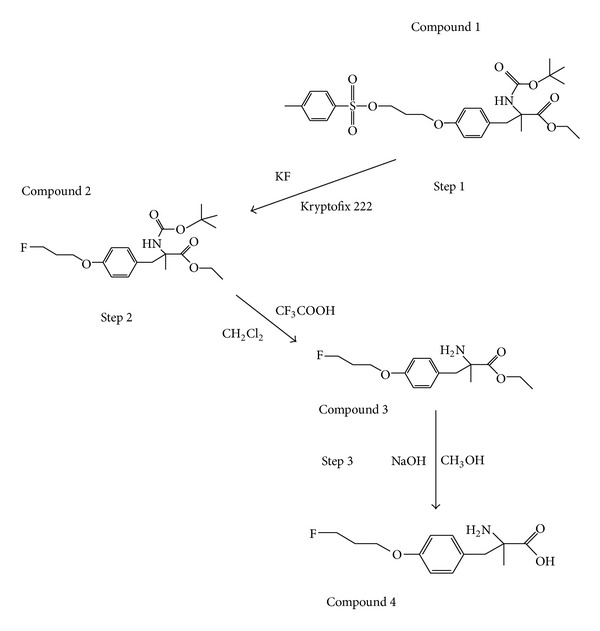
Synthetic scheme of FPAMT. The KF and kryptofix complex were incubated with N-t-butoxycarbonyl-O-[3-tosylpropyl]-*α*-methyl tyrosine ethyl ester (compound 1) in acetonitrile for synthesis of N-t-butoxycarbonyl-O-[3-^19^F-fluoropropyl]-*α*-methyl tyrosine ethyl ester (compound 2). After deprotection of butoxycarbonyl (BOC) of compound 2, O-[3-^19^F-fluoropropyl]-*α*-methyl tyrosine ethyl ester (compound 3) was synthesized. The final step is to yield FPAMT (compound 4) by deesterification of compound 3.

We used a three-step procedure to synthesize ^19^F-FPAMT (Figure 1). The first step was a displacement reaction. Kryptofix 222 (253.9 mg; 0.67 mmol) and K^19^F (40.5 mg; 0.69 mmol) were added to a vial containing N-t-butoxycarbonyl-O-[3-tosylpropyl]-*α*-methyl tyrosine ethyl ester (compound 1; 390 mg; 0.75 mmol) in acetonitrile (1 mL). The reaction vial was heated under reflux at 90°C for 40 min. After heating, the solution was evaporated to dryness. The mixture was reconstituted in 0.5 mL of ethyl acetate. N-t-butoxycarbonyl-O-[3-^19^F-fluoropropyl]-*α*-methyl tyrosine ethyl ester (compound 2) was purified by column chromatography using a silica gel column and eluted with hexane and ethyl acetate (4 : 1, v/v) to yield 120.0 mg of the compound. The second step was to deprotect butoxycarbonyl (BOC), and the third step was to remove ethyl ester groups. O-[3-^19^F-fluoropropyl]-*α*-methyl tyrosine ethyl ester (compound 3) was synthesized by reacting N-t-butoxycarbonyl-O-[3-^19^F-fluoropropyl]-*α*-methyl tyrosine ethyl ester (compound 2; 82.3 mg; 0.30 mmol) with trifluoroacetate (0.7 mL) in dichloromethane (2.0 mL) at room temperature for 50 min. After the solvent was evaporated to dryness, sodium hydroxide (1 N; 1.0 mL) in methanol (1.0 mL) was added, and the mixture was heated at 90°C for 15 min to remove ethyl ester group. The mixture was passed through a 0.22 *μ*M filter to yield ^19^F-FPAMT (compound 4). NMR and mass spectrometry were used to confirm the structure of this compound.

### 2.4. Manual Radiosynthesis of ^18^F-FPAMT

[^18^F]Fluoride in kryptofix complex (100 mCi in 0.3 mL acetonitrile) was purchased from the cyclotron facility of Cyclotope (Houston, TX, USA). N-t-butoxycarbonyl-O-[3-tosylpropyl]-*α*-methyl tyrosine ethyl ester (2 mg; 3.83 *μ*mol) dissolved in acetonitrile (0.1 mL) was added to the [^18^F]fluoride-kryptofix complex (51.5 mCi). The reaction mixture was heated at 90°C for 15 min to allow the displacement to occur. After the reaction mixture cooled, it was passed through a 500 mg silica gel packed SPE column (Whatman Lab., Clifton, NJ, USA) and eluted with acetonitrile (2 mL). The acetonitrile was then evaporated *in vacuo* at 85°C. The resulting mixture was hydrolyzed with trifluoroacetate (0.2 mL) in dichloromethane (0.2 mL) at room temperature for 10 min to deprotect BOC. After the solvent was evaporated to dryness *in vacuo*, sodium hydroxide (1 N; 0.2 mL) in methanol (0.2 mL) was added and heated at 90°C for 15 min to remove ethyl ester group. After methanol evaporated, hydrochloric acid (0.1 N; 0.2 mL) was used to adjust the pH of the final product to 6.5. Radio-TLC and HPLC were performed to assure the purity and identity of the product.

### 2.5. Automated Radiosynthesis of ^18^F-FPAMT

**Figure 2:**
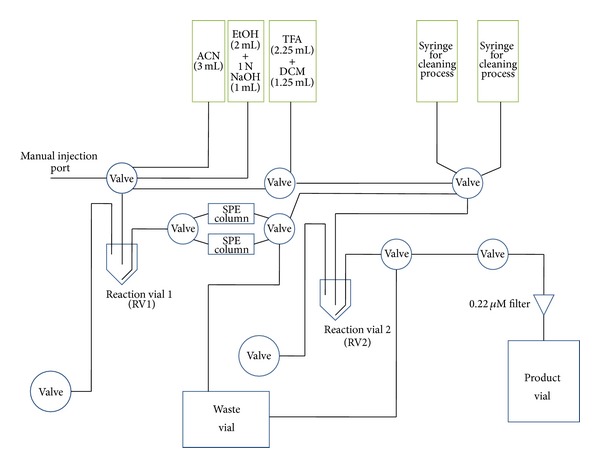
Diagram of this customized automated synthesis device. Radioisotope can be manually injected into reaction vial 1 (RV1) through the manual injection port. The upper five green blocks are syringes which were loaded with different chemicals for synthesis or cleaning process. Other blue blocks are fixed parts, such as valves, vials, and columns.

The automated radiosynthesis of ^18^F-FPAMT was achieved by our customized automated module. The diagram of this automated module is shown in Figure 2. The automated radiosynthesis consisted of three steps: nucleophilic substitution, deprotection of BOC, and deesterification. Before radiosynthesis was completed, the reaction vial 1 (RV1) was preloaded with N-t-butoxycarbonyl-O-[3-tosylpropyl]-*α*-methyl tyrosine ethyl ester (6.2 mg; 11.8 *μ*mol), and three syringes were loaded with different solutions: acetonitrile (3.0 mL), trifluoroacetate in dichloromethane (2.5 mL; 1 : 1, v/v), and sodium hydroxide in ethyl alcohol (1 N; 3.0 mL; 1 : 2, v/v). For the nucleophilic substitution, [^18^F]fluoride-kryptofix complex (0.2 mL; 29.36 mCi) was manually injected into the RV1 through the injection hole, and additional acetonitrile (0.35 mL) was manually injected into the RV1 to flush the residual [^18^F]fluoride-kryptofix complex inside the flow channel. Following this step, the infrared (IR) heater automatically heated the RV1 at 90°C for 15 min. For free fluoride separation, the mixture in the RV1 was automatically passed through a silica gel packed column (SPE 500 mg; Whatman Lab., Clifton, NJ, USA) to the reaction vial 2 (RV2) via nitrogen flow. Additional acetonitrile (2.0 mL) was then added to RV1, and the residual mixture was filtered through a SPE column to remove the free fluoride. The solution inside RV2 was evaporated *in vacuo* at 90°C for 15 min before deprotection of BOC was performed. Trifluoroacetate in dichloromethane (0.4 mL) was loaded into RV2, and the solution was set under room temperature for 10 min to allow the reaction to finish. The solvent was then evaporated to dryness *in vacuo* for 15 min. For deesterification, sodium hydroxide in methanol (0.6 mL) was loaded into RV2. The reaction mixture in RV2 was heated at 90°C for 15 min. Once deesterification was completed, the solvent in RV2 was evaporated *in vacuo*, and the radioactivities of the solvent in the column, RV1, and RV2 were measured upon the completion of ^18^F-FPAMT. Radio-TLC and HPLC were performed to assure the purity and identity of the final product.

### 2.6. *In Vitro* Cellular Uptake Studies

Rat mesothelioma IL-45 cells were maintained in the mixtures of Dulbecco's modification of Eagle's medium, F-12 (GIBCO, Grand Island, NY, USA), and 10% phosphate-buffered saline at 37°C in a humidified atmosphere containing 5% CO_2_. Cells were plated onto 6-well tissue culture plates (2 × 10^5^ cells/well) and incubated with ^18^F-FPAMT (8 *μ*Ci/well) or ^18^F-FDG (Cyclotope, Houston, TX, USA; 8 *μ*Ci/well) for 0–2 h. After incubation, the cells were collected, and their radioactivity was measured using a gamma counter. Data were expressed as the mean percent ± the standard deviation of the cellular uptake of ^18^F-FPAMT or ^18^F-FDG.

### 2.7. Biodistribution of ^18^F-FPAMT and ^18^F-FDG in Mesothelioma-Bearing Rats

Three hundred forty-four female Fischer rats (140–185 g) were obtained from Harlan, Inc. (Indianapolis, IN, USA). The rats were housed in an animal facility at The University of Texas MD Anderson Cancer Center. All protocols involving animals were approved by the Animal Use and Care Committee at MD Anderson Cancer Center. Nine rats were inoculated with mesothelioma IL-45 cells (1 × 10^5^ cells/rat) at the hinged leg. Twelve days after being inoculated with the mesothelioma cells, the rats were anesthetized with ketamine (10–15 mg/rat). ^18^F-FPAMT dissolved in saline (0.5 mCi/5 mL) was injected intravenously into 9 rats (*n* = 3 rats/group, 30 *μ*Ci/rat,). For comparison, the clinical standard, ^18^F-FDG (Cyclotope,), was injected intravenously into 9 rats (*n* = 3 rats/group; 30 *μ*Ci/rat). The distribution of ^18^F-FPAMT or ^18^F-FDG in various tissues was assessed at 30 min, 1.5 hrs, and 3 hrs after injection by COBRA. Percent of injected dose per tissue type was then calculated, and the data were expressed as the mean percent ± the standard deviation of the injected dose.

### 2.8. Dosimetry of ^18^F-FPAMT and ^18^F-FDG

Dosimetric calculations were performed from 30 to 180 min after the administration of ^18^F-FPAMT and ^18^F-FDG, and time-activity curves were generated for each organ. Analytic integration of the curves was used to determine the area under the curve (AUC), which was divided by the injected dose to yield the residence times of ^18^F-FPAMT and ^18^F-FDG in each organ. Residence times were then used to calculate target organ absorbed radiation doses based on the medical internal radiation dosimetry methodology for the normal adult male using the Olinda software package (Oak Ridge, TN, USA).

### 2.9. PET Imaging of Mesothelioma-Bearing Rats

Mesothelioma-bearing rats cells were imaged when their tumors were 1-2 cm in diameter. The rats were anesthetized with 2% isoflurane and administered with 500 *μ*Ci of ^18^F-FDG or 500 *μ*Ci of ^18^F-FPAMT. Four serial 15-minute transaxial PET images of each rat were obtained using microPET (Siemens Medical Systems, Inc., IL, USA).

## 3. Results

### 3.1. Chemistry

The synthetic schemes of ^18^F-FPAMT and ^19^F-FPAMT are shown in Figure 1. The structure of precursor N-t-butoxycarbonyl-O-[3-tosylpropyl]-*α*-methyl tyrosine ethyl ester (compound 1) was confirmed using ^1^H-NMR and mass spectrometry. The ^1^H-NMR (CDCl_3_) result was the following: *δ* = 7.76 (d, 2 H, *J* = 8.1 Hz), 7.26 (d, 2 H, *J* = 8.1 Hz), 6.97 (d, 2 H, *J* = 8.4 Hz), 6.67 (d, 2 H, *J* = 8.7 Hz), 4.23 (t, 2 H, *J* = 12.0 Hz), 4.12 (q, 2 H, *J* = 7.2 Hz, *J* = 7.2 Hz), 3.92 (t, 2H, *J* = 11.7 Hz), 3.22 (q, 2H, *J* = 13.5 Hz, *J* = 12.9 Hz), 2.40 (s, 3 H), 2.12 (m, 2 H), 1.54 (s, 3 H), 1.47 (s, 9 H), and 1.29 (t, 3 H, *J* = 12.3 Hz) ppm; M/Z: 558.29 (M+Na)^+^.

^19^F-FPAMT was obtained after subjecting compound 1 to nucleophilic substitution, free fluoride separation, deprotection of BOC, and deesterification. The structure of ^19^F-FPAMT (compound 4) was confirmed using ^1^H-NMR and mass spectrometry. The ^1^H-NMR (D_2_O) result the following result was: *δ* = 7.17 (d, 2 H, *J* = 8.4 Hz), 6.93 (d, 2 H, *J* = 8.7 Hz), 4.75 (t, H, *J* = 11.7 Hz), 4.59 (t, H, *J* = 11.7 Hz), 4.13 (t, 2 H, *J* = 12.3* *Hz), 2.84 (dd, *J* = 13.2* *Hz, *J* = 13.5* *Hz), 2.14 (m, 2 H), and 1.29 (s, 3 H) ppm. ^19^F-NMR *δ* = 220.33; M/Z: 406.38 (M+Na)^+^.

### 3.2. Radiochemistry

The ^18^F-displacement reaction produced 35.4 mCi (yield: 78%, decay corrected) of N-t-butoxycarbonyl-O-[3-^18^F-fluoropropyl]-*α*-methyl tyrosine ethyl ester, and the residual in the column was 3.77 mCi (8.3%, decay corrected). The no-carrier-added displacement product corresponded to the unlabeled N-t-butoxycarbonyl-O-[3-fluoropropyl]-*α*-methyl tyrosine ethyl ester under the same TLC system (hexane : ethyl acetate; 10 : 3, v/v) and HPLC system (20 *μ*L loop, 210 nm, Bondapak CN-RP column, Waters, eluted with methanol : water, 3 : 2, v/v; flow rate 1.0 mL/min). The retention factor (*R*_*f*_) of N-t-butoxycarbonyl-O-[3-^18^F-fluoropropyl]-*α*-methyl tyrosine ethyl ester was 0.46 with purity >99%. Under the same conditions, the *R*_*f*_ value for [^18^F]fluoride in kryptofix complex was 0.1. After hydrolysis, ^18^F-FPAMT stayed at origin (*R*_*f*_ = 0.1). The retention times for N-BOC and the ethyl ester form of tosylpropyl-, fluoropropyl-, and ^18^F-fluoropropyl-*α*-ethyltyrosine were 16.13, 8.37, and 8.79 min, respectively. The decay-corrected yield for hydrolysis (deprotection of BOC and deesterification) was 89%. At the end-of-synthesis (88 min), 10 mCi of ^18^F-FPAMT was obtained, and the decay-corrected yield was 34%. The specific activity of this compound was 0.32 Ci/*μ*mol. For the automated synthesis of ^18^F-FPAMT, the decay-corrected yield was 15%, the end-of-synthesis time was 110 min, and the specific activity was 0.16 Ci/*μ*mol.

### 3.3. *In Vitro* Cellular Uptake Studies

**Figure 3:**
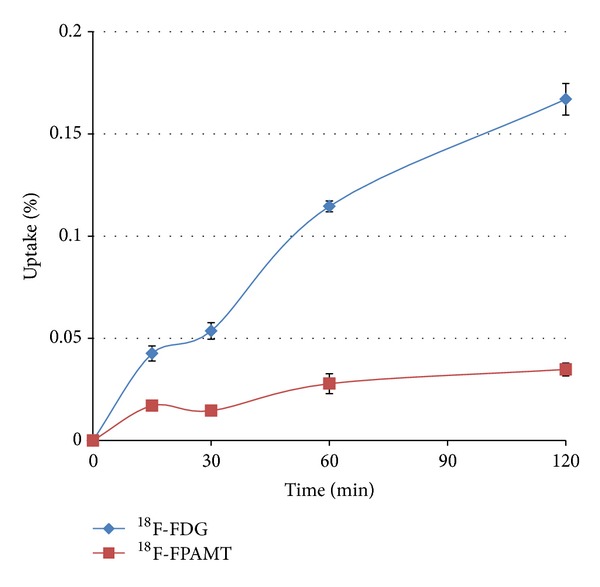
*In vitro* cellular uptake of ^18^F-FPAMT and ^18^F-FDG in mesothelioma cells (IL-45). Data are expressed as mean percent of cellular uptake ± standard deviation (%uptake ± SD) measured at 15, 30, 60, and 120 min.

The uptake of ^18^F-FPAMT reached saturation at 60 min (Figure 3). ^18^F-FDG uptake continued to increase throughout the period, and the percentage uptake of ^18^F-FDG was higher than that of ^18^F-FPAMT at each time point.

### 3.4. Biodistribution of ^18^F-FPAMT and ^18^F-FDG in Mesothelioma-Bearing Rats

The distributions of ^18^F-FPAMT and ^18^F-FDG in various tissues in mesothelioma-bearing rats are shown in Tables 1 and 2, respectively. Both compounds showed no marked increase in bone uptake, representing their *in vivo* stability. High kidney and pancreas uptake of ^18^F-FPAMT was observed, and this phenomenon was also observed from other tyrosine-based radiotracers [18]. Unlike ^18^F-FDG, ^18^F-FPAMT had poor uptake in brain tissue.

### 3.5. Dosimetry of ^18^F-FPAMT in Rats

The estimated absorbed radiation dose of ^18^F-FPAMT is shown in Table 3. According to the US Food and Drug Administration Regulations, human exposure to radiation from the use of “radioactive research drugs” should be limited to 3 rem per single administration and 3 rem per year to the whole body, blood-forming organs (red marrow, osteogenic cells, and spleen), the lens of the eye, and gonads (testes and uterus); the limit for other organs is 5 rem per single administration and 15 rem annually. The total rem of ^18^F-FPAMT absorbed by each organ was below these limits at the proposed injection of 30 mCi per patient.

### 3.6. Imaging of Mesothelioma-Bearing Rats

**Figure 4:**
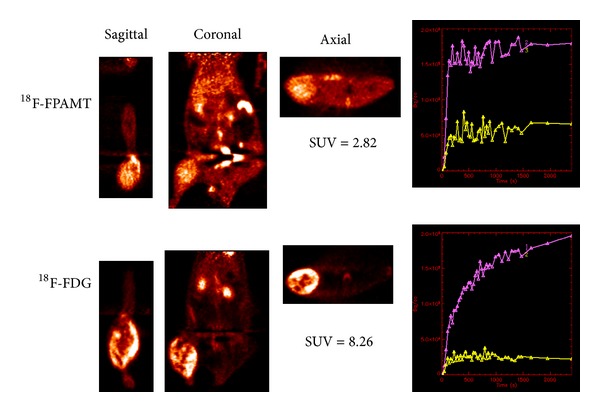
*μ*PET images of ^18^F-FPAMT and ^18^F-FDG in mesothelioma-bearing rats (lower body: IL-45, at 45 min). The SUV ratios of tumor to muscle for ^18^F-FPAMT and ^18^F-FDG were 2.82 and 8.26, respectively. Computer-outlined regions of interest (ROI) (counts per pixel) for tumor and muscle at the corresponding time interval were used to generate a dynamic plot. Dynamic plot was from 0 to 45 minutes.

**Figure 5:**
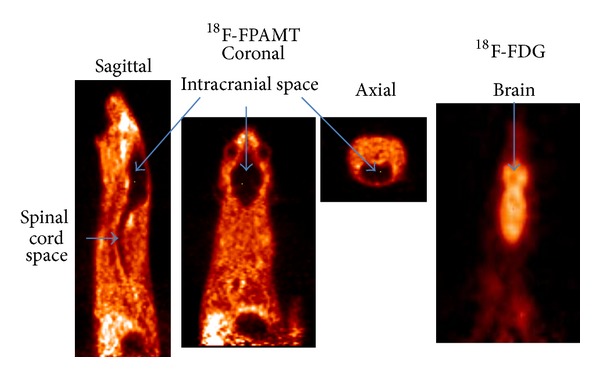
*μ*PET images of ^18^F-FPAMT and ^18^F-FDG in mesothelioma-bearing rats (upper body: IL-45, at 45 min). There was extremely low uptake of ^18^F-FPAMT in the brain and spinal cord when compared with ^18^F-FDG.

Scintigraphic images of mesothelioma-bearing rats administrated ^18^F-FPAMT or ^18^F-FDG showed that tumors could be clearly detected, and bone uptake was low (Figure 4). The standardized uptake value (SUV) curve of ^18^F-FPAMT for tumor and muscle reached the plateau at 30 min after injection, but the SUV curve of ^18^F-FDG for tumor continued increasing during the imaging. The SUV ratios of tumor to muscle for ^18^F-FPAMT and ^18^F-FDG were 2.82 and 8.26, respectively. There was extremely low uptake of ^18^F-FPAMT in the brain and spinal cord when compared with ^18^F-FDG (Figure 5).

## 4. Discussion

Mesothelioma is an asbestos-related neoplasm generating from mesothelial cells in the pleural, peritoneal, and pericardial cavities, and its incidence increased in several countries [19]. The diagnostic tools and treatment regimens for these tumors are disappointing, and median survival time is 12 months after initial diagnosis [20]. The initial diagnoses of mesothelioma are based on patient's medical history and physical examination. After that, computed tomography scans and magnetic resonance imaging are used to screen patients, and then biopsy test is needed to confirm the incidence of mesothelioma. ^18^F-FDG/PET scan is the tool to determine whether a suspicious area is malignant mesothelioma or a benign condition such as pleural scarring, and the result can identify the best area for an accurate biopsy. PET scans are also effective for highlighting mesothelioma metastases that may not appear on other conventional imaging scans. However, ^18^F-FDG/PET scans have limitations in differential diagnosis between cancerous cells and inflammation tissues which metabolize glucose with abnormally high rates. In this case, radiolabeled amino acids are the alternative methods to detect malignant pleural mesothelial and other cancerous cells which overexpress unregulated amino acid transporters [21–23]. Mesothelioma rat model was then selected because rat model provided better anatomical differentiation than mouse model in imaging studies. It is more accurate to determine radiation dosimetry from biodistribution data.

^18^F-FET and ^18^F-FAMT are radiolabeled amino acids, and they are useful in imaging cancers. However, existing methods for synthesizing these compounds result in low yields, thus limiting the availability of ^18^F-FET and ^18^F-FAMT in the clinic. In the present study, we synthesized ^18^F-FPAMT, an ^18^F-FAMT analog, and used a mesothelioma rat model to preliminarily evaluate it as a tumor-imaging compound. We used NMR and mass spectrometry to confirm the structure of ^19^F-FPAMT. The yield of ^19^F-FPAMT was 46.71%. N-t-butoxycarbonyl-O-[3-tosylpropyl]-*α*-methyl tyrosine ethyl ester was used as the starting material for manual and automated syntheses of ^18^F-FPAMT. The quality control of ^18^F-FPAMT was evaluated by radio-TLC and HPLC. Manual synthesis of ^18^F-FPAMT resulted in the decay-corrected yield of 34%, radiochemical purity of >95%, the specific activity of 0.32 Ci/*μ*mol, and pH value of 5 to 6; the manual synthesis time was 88 min. Automated synthesis of ^18^F-FPAMT resulted in the decay-corrected yield of 15%, radiochemical purity of >95%, the specific activity of 0.16 Ci/*μ*mol, and pH value of 5 to 6; the manual synthesis time was 110 min.

The traditional method of radiosynthesizing ^18^F-labeled tyrosine analogs such as ^18^F-FET and ^18^F-FAMT was through electrophilic substitution reaction which has low synthetic yield. Besides, the reaction uses ^18^F-F_2_ gas, and HPLC separation makes it even difficult to use this method in automated modules. Although a nucleophilic reaction could result in a high yield of ^18^F-FET (40%), this method still requires HPLC for purification, and, thus, it is not ideal to use this synthesis method in automated synthesis modules. In the present study, we obtained ^18^F-FPAMT by a nucleophilic reaction, but we completed the purification process without HPLC. Therefore, our method of synthesizing ^18^F-FPAMT can be applied to the customized automated synthesis module.

For the *in vitro* studies, although the result showed that ^18^F-FPAMT had lower cellular uptake than that of ^18^F-FDG, the uptake mechanism of these two compounds is different. Malignant cells utilize ^18^F-FDG as glucose for upregulated aerobic glycolysis and ^18^F-FPAMT as an amino acid for proliferation. The results indicate that ^18^F-FPAMT has the potential to become a tumor detecting tracer. Biodistribution studies showed that ^18^F-FPAMT and ^18^F-FDG were rapidly cleared from blood and distributed in other tissues. Compared with ^18^F-FDG, the accumulation of ^18^F-FPAMT was significantly lower in heart, lungs, thyroid, spleen, and brain. High accumulation of ^18^F-FPAMT was observed in the kidneys and pancreas after administration. This could be due to the high expression of the amino acid transporters in the kidneys and pancreas [4]. These results were consistent with those of other radiolabeled amino acid analogs such as ^18^F-FAMT [24] and ^77^Br-BAMT [18], although ^18^F-FET showed only higher uptake in kidneys [25]. The bone uptakes of ^18^F-FPAMT and ^18^F-FDG at 180 min after administration increased slightly, suggesting defluorination of both compounds. In the microPET studies of ^18^F-FDG and ^18^F-FPAMT, the lesions could be observed clearly at 45 min after administration (Figure 4), and the accumulation of ^18^F-FPAMT in the brain and spinal cord was significantly less than that of ^18^F-FDG (Figure 5), suggesting that ^18^F-FPAMT has great potential in imaging brain tumors.

## 5. Conclusion

In this study, we manually synthesized ^18^F-FPAMT with high yielding and radiochemical purity, and we used the customized automated synthesizer for the proof of concept of automated manufacturing of ^18^F-FPAMT. Both *in vitro* and *in vivo* studies suggested that ^18^F-FPAMT can be a good PET agent for detecting mesothelioma, and it might have great potential in brain tumor imaging. In the future, we will focus on optimization of the automated processes for a better yield and a higher specific activity.

## Figures and Tables

**Table 1:** Biodistribution of ^18^F-FPAMT in rats.

% of injected dose per gram of tissue weight (*n* = 3/time, interval (iv))			
	30 min	90 min	180 min
Blood	0.37 ± 0.07	0.11 ± 0.01	0.04 ± 0.00
Heart	0.32 ± 0.04	0.12 ± 0.01	0.04 ± 0.00
Lungs	0.31 ± 0.06	0.09 ± 0.01	0.03 ± 0.00
Thyroid	0.28 ± 0.01	0.14 ± 0.01	0.09 ± 0.01
Pancreas	0.84 ± 0.11	0.19 ± 0.03	0.07 ± 0.01
Liver	0.49 ± 0.06	0.14 ± 0.01	0.05 ± 0.00
Spleen	0.34 ± 0.05	0.09 ± 0.01	0.03 ± 0.00
Kidneys	3.86 ± 0.74	0.90 ± 0.13	0.40 ± 0.02
Stomach	0.27 ± 0.03	0.09 ± 0.01	0.03 ± 0.00
Intestines	0.37 ± 0.04	0.15 ± 0.05	0.04 ± 0.00
Uterus	0.27 ± 0.03	0.07 ± 0.01	0.03 ± 0.00
Muscle	0.27 ± 0.03	0.15 ± 0.01	0.07 ± 0.01
Bone	0.11 ± 0.01	0.10 ± 0.02	0.19 ± 0.05
Brain	0.02 ± 0.00	0.01 ± 0.00	0.01 ± 0.00

Values shown represent the mean ± standard deviation of data from 3 animals.

**Table 2:** Biodistribution of ^18^F-FDG in rats.

% of injected dose per gram of tissue weight (*n* = 3/time, interval (iv))			
	30 min	90 min	180 min
Blood	0.45 ± 0.07	0.15 ± 0.01	0.07 ± 0.01
Heart	3.42 ± 1.14	1.95 ± 0.40	1.94 ± 0.45
Lungs	0.60 ± 0.07	0.53 ± 0.03	0.46 ± 0.06
Thyroid	0.65 ± 0.04	0.47 ± 0.05	0.54 ± 0.04
Pancreas	0.22 ± 0.02	0.21 ± 0.02	0.21 ± 0.03
Liver	0.51 ± 0.08	0.33 ± 0.03	0.23 ± 0.03
Spleen	0.88 ± 0.08	0.87 ± 0.06	0.98 ± 0.10
Kidneys	0.85 ± 0.13	0.43 ± 0.04	0.23 ± 0.01
Stomach	0.55 ± 0.03	0.40 ± 0.03	0.38 ± 0.02
Intestines	0.94 ± 0.16	1.00 ± 0.22	0.62 ± 0.07
Uterus	0.52 ± 0.06	0.57 ± 0.08	0.39 ± 0.09
Muscle	0.45 ± 0.14	0.23 ± 0.03	0.42 ± 0.06
Bone	0.21 ± 0.09	0.14 ± 0.07	0.24 ± 0.06
Brain	2.36 ± 0.10	2.24 ± 0.20	1.89 ± 0.35

Values shown represent the mean ± standard deviation of data from 3 animals.

**Table 3:** Radiation dose estimates of reference adult for ^18^F-FPAMT.

Target organ	rad/mCi	human dose (mCi)	rad
Organs (5 rem annually/15 rem total)			
Adrenals	2.98*E* − 03	30	0.089
Brain	9.27*E* − 04	30	0.028
Breasts	1.95*E* − 03	30	0.059
Gall bladder wall	2.88*E* − 03	30	0.086
Lli wall	3.16*E* − 03	30	0.095
Small int.	3.54*E* − 03	30	0.106
Stomach	2.79*E* − 03	30	0.084
Uli wall	3.21*E* − 03	30	0.096
Heart wall	2.94*E* − 03	30	0.088
Kidneys	6.19*E* − 03	30	0.186
Liver	1.51*E* − 03	30	0.045
Lungs	2.40*E* − 03	30	0.072
Muscle	1.63*E* − 03	30	0.049
Pancreas	3.38*E* − 03	30	0.101
Bone surfaces	6.88*E* − 03	30	0.206
Skin	1.55*E* − 03	30	0.047
Testes	2.29*E* − 03	30	0.069
Thymus	2.43*E* − 03	30	0.073
Thyroid	2.47*E* − 03	30	0.074
Urine bladder wall	3.01*E* − 03	30	0.090
Uterus	3.36*E* − 03	30	0.101
Eff dose	2.61*E* − 03	30	0.078

Blood-forming organs (3 rem annually/5 rem total)			
Ovaries	3.24*E* − 03	30	0.097
Red marrow	2.29*E* − 03	30	0.069
Spleen	3.42*E* − 03	30	0.103
Eff dose eq.	3.14*E* − 03	30	0.094
Total body	2.35*E* − 03	30	0.071
